# The ADP-Ribosylating Toxins of *Salmonella*

**DOI:** 10.3390/toxins11070416

**Published:** 2019-07-16

**Authors:** Rachel A. Cheng, Martin Wiedmann

**Affiliations:** Department of Food Science, Cornell University, Ithaca, NY 14853, USA

**Keywords:** ADP-ribosyltransferase, toxin, Salmonella, SpvB, ArtAB, typhoid toxin, SboC, SeoC

## Abstract

A number of pathogenic bacteria utilize toxins to mediate disease in a susceptible host. The foodborne pathogen *Salmonella* is one of the most important and well-studied bacterial pathogens. Recently, whole genome sequence characterizations revealed the presence of multiple novel ADP-ribosylating toxins encoded by a variety of *Salmonella* serovars. In this review, we discuss both the classical (SpvB) and novel (typhoid toxin, ArtAB, and SboC/SeoC) ADP-ribosylating toxins of *Salmonella*, including the structure and function of these toxins and our current understanding of their contributions to virulence.

## 1. Introduction

*Salmonella* is one of the most diverse and successful pathogens. Although the genus *Salmonella* includes just two species, *enterica* and *bongori*, there are 2,659 different serovars [1]. A total of 6 subspecies are included within species *enterica*: (I) *enterica*, (II) *salamae*, (IIIa) *arizonae,* (IIIb) *diarizonae*, (IV) *houtenae*, and (VI) *indica* [1,2]. As the majority of animal and human clinical cases result from infection with *S. enterica*—although infections with *S. bongori* are occasionally reported [3]—research efforts have primarily focused on the characterization of *S. enterica*, and more specifically on *S. enterica* subsp. *enterica* (abbreviated here as “*S.*” followed by the serovar). Salmonellae are further classified based on the disease that they cause. While the majority of serovars cause a mild gastroenteritis (generally caused by nontyphoidal *Salmonella* [NTS] serovars), a select few (Typhi, Sendai, and Paratyphi A, B, or C) cause an invasive, severe infection known as enteric fever [4]. Exceptions to this generalization exist, as some NTS serovars, such as Dublin and Choleraesuis, are frequently associated with invasive disease [5].

Each year, *Salmonella* causes an estimated 88 million combined illnesses (including NTS, Paratyphi, and Typhi) worldwide [6]. The burden of salmonellosis varies by geographical region [7], as do the specific serovars that are associated with human clinical disease in those regions [8,9]. *Salmonella* is also an important cause of animal disease, as infection with some serovars is associated with severe clinical disease in select animals, such as *S.* Gallinarum in chickens [10] and *S.* Dublin in cows [11]. Although animals can be colonized by *Salmonella*, and direct contact with animals is a risk factor for salmonellosis [12], the vast majority of infections are foodborne [13] and are often the result of sporadic infection (i.e., not direct contact with animals) [14].

*S.* Typhimurium, although somewhat serendipitously identified based on its ability to cause mouse typhoid, has become the model for studying NTS serovars [15]. *S.* Typhi has also been extensively characterized, due to the severity of disease that it causes [16]. As such, conclusions about nontyphoidal and typhoid *Salmonella* are generally extrapolated from studies with *S.* Typhimurium and *S.* Typhi. In contrast to other pathogens that cause a toxin-mediated disease, nontyphoidal salmonellosis is largely the result of the proinflammatory reactions triggered by interactions with the host’s immune system. *Salmonella* uses a T3SS encoded within *Salmonella* pathogenicity island (SPI) 1 to secrete effectors into host cells, resulting in membrane ruffling and subsequent engulfment of *Salmonella*. Once inside a host cell, the activation of SPI-2 genes allows *Salmonella* to survive and multiply. This allows NTS serovars to promote an inflammatory response in the host’s intestine, resulting in the generation of alternate terminal electron acceptors, thereby enabling NTS to compete with the resident anaerobic bacteria of the gut microbiota [17]. In contrast, *S.* Typhi utilizes a more stealth approach by (i) expressing a Vi capsular antigen that is associated with reductions in IL-8 expression of both immune and epithelial cells [18], (ii) down-regulating flagellin, resulting in reduced pyroptosis [19], a pro-inflammatory form of cell death, and (iii) producing a novel toxin (typhoid toxin), which has been shown to reduce the levels of circulating immune cells in the blood [20]. Our current understanding about the dichotomous pathogenesis of typhoid and nontyphoidal serovars illustrates the importance of the immune response in creating an inflammatory environment in which nontyphoidal serovars can excel and an environment in which Typhi and other invasive serovars can hide.

ADP-ribosylating toxins play an important role in pathogenesis for a number of bacterial pathogens, including *Bordetella pertussis* [21], *Clostridium botulinum* [22], *Corynebacterium diphtheriae* [23], *Vibrio cholerae* [24], and others [25,26]. Despite having a common activity (ADP-ribosylation of target proteins), bacterial ADP-ribosylating toxins (bARTTs [25]) target a wide range of host proteins, therefore resulting in a variety of cytotoxic effects, ranging from modulation of the cytoskeleton to promoting host cell death. These toxins constitute two general classes: (i) the diphtheria toxin-like (DT-like) group composed of an active (A) and binding (B) domain present in a single chain (AB), and (ii) the cholera toxin-like (CT-like) group, composed of a single A domain that is non-covalently bound to a pentameric subunit of 5 B domains (AB_5_) [25]. CT-like toxins can further be categorized into C-2-like binary toxins, in which the A-domain and B-domain subunits are expressed and synthesized separately [25], and C3-like exoenzymes consisting of a single A-domain subunit [25]. Recently, a number of novel bARTTs were discovered and characterized [20,27,28], expanding the diversity of this toxin family [25].

While salmonellosis is not traditionally considered a toxin-mediated disease, certain strains of *Salmonella* utilize bARTTs (Table 1) to alter host responses in order to promote pathogenesis in their respective hosts. SpvB, perhaps the most well-known of these toxins encoded by *Salmonella*, is a C2-like bARTT that ADP-ribosylates actin monomers, preventing their polymerization [29]. Three novel bARTT’s—the typhoid toxin [30], ArtAB [31], and SboC/SeoC [32]—were only recently discovered, and will be discussed in further detail in this review. Interestingly, the typhoid toxin produced by *S.* Typhi was reported to recapitulate many of the clinical signs of typhoid fever in a mouse model [20]. However, recent genomic characterizations of a wide range of nontyphoidal and paratyphoidal serovars revealed the presence of this toxin in many different serovars, most of which do not cause typhoid-like disease in humans, therefore suggesting that the typhoid toxin likely contributes to virulence but is not the sole driving force of typhoid fever.

In this review, we discuss the current knowledge regarding the bARTT’s produced by *Salmonella* serovars, and our current understanding of how these toxins contribute to *Salmonella’s* success as a pathogen.

## 2. SpvB Toxin

**Figure 1:**
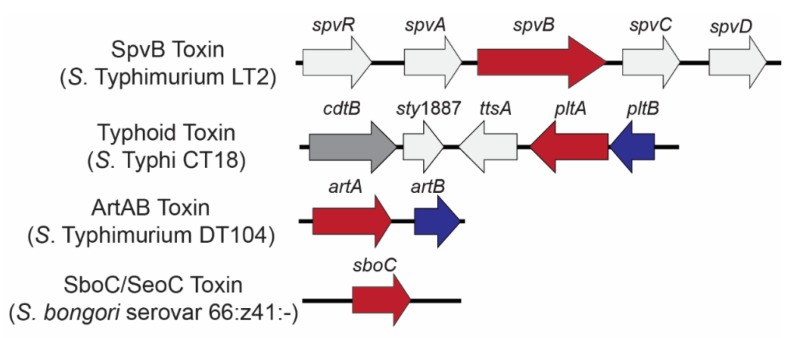
Genetic loci encoding *Salmonella* ADP-ribosylating toxins. Genes are color coded to represent toxin components: A domain-encoding (ADP-ribosyltransferase) genes (red) and B (binding) domain-encoding genes (blue); the genes colored light gray are co-located with bARTT genes but are not part of the final holotoxin. Typhoid toxin has an additional A subunit (CdtB; dark gray) which acts as a nuclease.

The presence of a high molecular weight plasmid encoded by *S.* Typhimurium isolates was initially reported in the 1970s, although the plasmid was initially characterized as ‘cryptic’ and was solely associated with its observed incapability with the F plasmid [38]. It was later discovered that this ‘cryptic’ plasmid was associated with enhanced adhesion and invasion of HeLa cells, and pathogenesis in mice [39]. Cloning of random fragments from endonuclease digests identified a 7.8 kb segment essential for virulence in plasmid-cured strains of *S.* Dublin and *S.* Typhimurium [40], which was later identified and named *spvRABCD* for *Salmonella* plasmid virulence (Figure 1) [38]. Further characterization demonstrated that SpvB functions as an ADP-ribosyltransferase that catalyzes ADP-ribosylation of host actin monomers, leading to their depolymerization [41]. *spv* genes have been identified in some *S. enterica* subsp. *arizonae* strains as well, but these genes are chromosomally-encoded and lack *spvD* [42].

Virulence plasmids from at least 10 different serovars have been identified as encoding *spv* genes (Table 2). It is important to note that these virulence plasmids also carry other virulence factors, including plasmid-encoded fimbriae (*pef*) and *rcK* (resistance to complement killing), as well as antimicrobial resistance genes [43]. Among the *spv* genes, *spvC* encodes a phosphothreonine lyase that inhibits host MAP kinases, [44] and *spvD* is associated with the suppression of proinflammatory responses [45].

### 2.1. SpvB Toxin Structure and Activity

**Figure 2:**
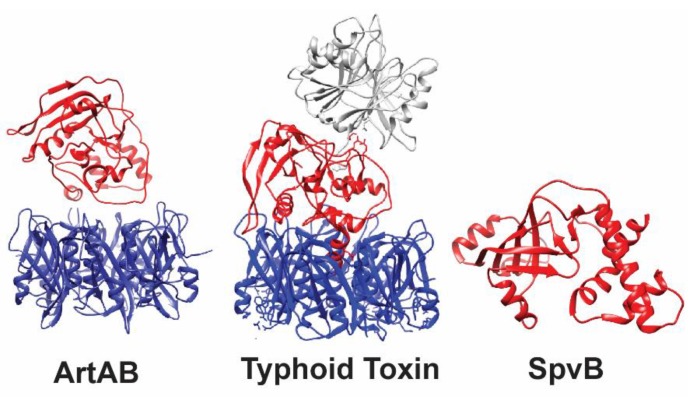
3-D Structures of ADP-ribosylating toxins of *Salmonella*. ArtAB and typhoid toxin are secreted exotoxins, while SpvB is translocated directly into the cytoplasm of host cells, and therefore lacks a binding domain. Active domains are shown in red, binding domains in blue, and other toxin components are shown in gray (typhoid toxin CdtB). The typhoid toxin (PDB accession: 4K6L) and SpvB (PDB accession: 2GWL) crystal structures were solved; the crystal structure of ArtA (PDB accession: 4Z9C; used as a template for modeling DT-104 ArtA) and the ArtB pentamer (PDB accession: 5WHV) were resolved as independent subunits. Therefore, the A and B domains of ArtAB are shown as two separate subunits and not as the final conformation of the toxin.

In contrast to other known actin-ADP-ribosylating toxins, SpvB is a single-chain A-domain toxin (i.e., it lacks a binding subunit, as seen in Figure 2) [25]. SpvB is secreted by intracellular *Salmonella* directly into the cytoplasm of host cells via the SPI-2 T3SS [56], where it catalyzes the mono-ADP-ribosylation of the Arg177 residue of actin monomeric subunits, thereby inhibiting actin’s ability to hydrolyze ATP, which prevents actin polymerization and ultimately results in the depolymerization of actin [36]. This activity opposes the role of several SPI-1- and SPI-2-encoded virulence factors, whose roles are to encourage the polymerization of actin for successful invasion in non-phagocytic epithelial cells, and to maintain the integrity of the SCV membrane, respectively [57,58].

### 2.2. Implications of SpvB-Mediated Salmonella Virulence

SpvB-mediated actin depolymerization is associated with an accumulation of cells in the G_2_/M phase, eventually resulting in apoptotic cell death [59]. As inhibition of actin polymerization may also cause arrest in the G_1_ phase [60], it is likely that SpvB’s activity also prolongs entry into, if not prevents entry into the G_2_ phase, although previous studies have not conclusively shown this. SpvB also inhibits vacuole-associated actin polymerization (VAP) [61], which plays an important role in maintenance of the SCV membrane [62]. This suggests that SpvB-mediated inhibition of actin depolymerization is associated with a breakdown of the SCV membrane, followed by escape of *Salmonella* into the host cytosol [61]. This is in contrast to the current understanding that *Salmonella* multiply and reside within the protective environment of the SCV [58], and instead suggests that SpvB mediates an intra-cytosolic *Salmonella* lifestyle more akin to that of other pathogens such as *Listeria monocytogenes* [63], *Shigella* spp. [64], and *Rickettsia* spp. [65]. Characterizations of the intracellular growth of SpvB-producing *Salmonella* will be important for understanding the implications of cytosolic *Salmonella*. Assessment of the proportion of SpvB-producing *Salmonella* cells that escape the SCV would provide important insight into the role of SpvB in modulating *Salmonella*’s intracellular lifestyle.

The primary observation that the *spv* locus plays an important role in the severity of disease stems from the fact that a number of *spv*-encoding serovars cause systemic disease in their respective hosts. With the exception of serovars Bovismorbificans, Enteritidis, and Typhimurium, most of the serovars that encode *spv* genes are host adapted (i.e., Choleraesuis to pigs [66], Dublin to cows [11], Abortusequi to horses and donkeys [67], and Abortusovis to sheep and goats [68]), or host restricted (i.e., Gallinarum to chickens [10] and Paratyphi C and Sendai to humans [53]). In general, infections with these serovars either in the hosts which they have adapted to, or in humans, often results in a more severe infection characterized by invasive disease [5]. An important exception to this is *S.* Gallinarum, which is restricted to chickens [10]. As a number of studies characterized the role of the virulence plasmid in pathogenesis, and not the role of *spvB*, conclusions drawn about SpvB’s specific role in pathogenesis should be considered in the context of the other plasmid-encoded virulence factors, especially considering that previous studies have shown that *spvBC*, but not *spvB* alone, is sufficient for replacing the entire virulence plasmid during infection of BALB/c mice, suggesting that both SpvB and SpvC are essential for mediating the toxin-associated phenotype in vivo [69]. One study used a mouse model to show that SpvB was required for efficient colonization of the intestinal lamina propia, and although SpvB and SpvC were not essential, they were associated with increased gut inflammation [37]. Another study demonstrated that deletion of *spvB* in *S*. Typhimurium resulted in a decrease in bacterial levels in the spleens of infected mice, but only at 14 days post-infection [70], suggesting that *spvB* may play a role in the systemic spread, or in persistence, but it is unlikely to affect the development of acute salmonellosis. In general, our understanding of SpvB’s role in virulence (based primarily on tissue culture models) has not been translated into obvious roles in pathogenesis, aside from a select few studies that demonstrated a potential role for SpvB in colonization and persistence in vivo, phenotypes that could be associated with other virulence factors that are encoded on the same virulence plasmid as the *spv* genes (i.e., the *pef* operon).

## 3. The Typhoid Toxin

The typhoid toxin, so named because it was originally identified in *S.* Typhi [30,71], is a novel A_2_B_5_ toxin that incorporates the nuclease activity of CdtB from the cytolethal distending toxin [72] with the ADP-ribosyltransferase activity from the pertussis toxin’s active subunit, PtxA (also called S1; Figure 2). The typhoid toxin is chromosomally encoded on a putative mobile element within SPI-11 [30,47] and includes the accessory genes *ttsA* and *STY1887* (Figure 1). While *ttsA* is predicted to play a role in typhoid toxin secretion, a role for *STY1887* has yet to be defined [73].

The vast majority of characterizations have focused on the DNase activity of the typhoid toxin, primarily because the Dnase activity of CdtB is associated with DNA damage. Furthermore, mutations at the catalytic site of CdtB are sufficient to abolish the cell cycle arrest associated with this toxin [20,30]. Although first characterized in *S.* Typhi, genes encoding the typhoid toxin have since been identified in at least 48 different serovars [33,47], including both typhoidal and nontyphoidal serovars, as well as in *S. bongori* (Table 2). A few studies have demonstrated the high conservation of PltA (average of 98–100% amino acid identity) across different serovars [52,74].

### 3.1. Typhoid Toxin Structure and ADP-Ribosyltransferase Activity

The typhoid toxin was originally characterized as utilizing a homopentameric binding subunit of PltB monomers [20], however new evidence suggests that the typhoid toxin can also form a functional holotoxin with a homopentameric binding subunit of ArtB monomers [75,76]. As ArtB and PltB preferentially bind to different sialoglycans on host cells, the use of multiple binding subunits of this toxin was proposed to enable the typhoid toxin to target different tissue types in humans [77], as well as different hosts [76]. X-ray crystallography of purified typhoid toxin revealed that CdtB and PltA, the two active subunits of the toxin, are tethered together by a disulfide bond between PltA’s Cys214 and CdtB’s Cys269 residues [20]. An α-helix at PltA’s C-terminus mediates the PltA–PltB association and is stabilized by interactions of six PltA amino acid residues with the hydrophobic lumen of the PltB homopentameric binding subunit, forming a stable pyramid-shaped toxin [20]. Interestingly, PltA shares homology with both PtxA from the pertussis toxin [30], and ArtA encoded by a number of different *Salmonella* serovars [33].

**Figure 3:**
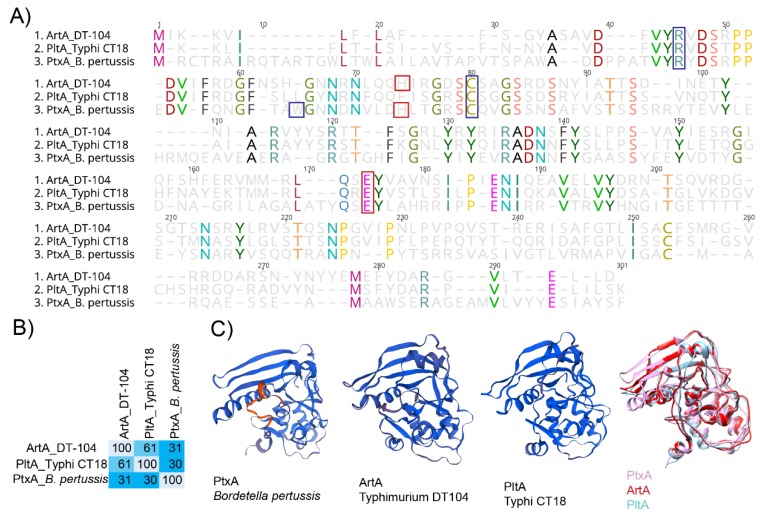
PltA and ArtA are structurally similar to the active subunit PtxA from the pertussis toxin. (**A**) Alignment of predicted amino acid sequence of ArtA from *S.* Typhimurium DT104, PltA from *S.* Typhi CT18, and PtxA from *B. pertussis*. Red boxes indicate catalytic residues, and blue boxes represent amino acid residues involved in NAD^+^ binding. (**B**) Percent conservation of amino acids in ArtA, PltA, and PtxA; calculations were performed using Geneious software. (**C**) Structure of ADP-ribosylating subunits for PtxA (PDB accession: 1PRT), ArtA (modeled onto 479C), PltA (4K6L), and all three. Alignment of 3D models was done with Chimera software [79].

Intracellularly, host cell reductases in the endoplasmic reticulum reduce the CdtB–PltA disulfide bond, releasing CdtB from the rest of the holotoxin [78]; hypothetically, the Cys56-Cys207 bond in PltA [20] is also reduced, thereby enabling PltA to bind NAD^+^ [20], and subsequently mono-ADP ribosylate target host proteins in a manner similar to that of other bARTTs, including the pertussis toxin [25]. PltA is able to ADP-ribosylate proteins in extracts from cultured epithelial cells [30], although the target of this activity has yet to be identified. Given its structural homology and the conservation of amino acid residues involved in both NAD^+^ binding and the catalytic activity of PtxA from the pertussis toxin (Figure 3) [20], the target of PltA mono-ADP-ribosylation may be host heterometric G-proteins, although this has yet to be definitively confirmed.

### 3.2. Implications of PltA-Mediated Salmonella Virulence

While the administration of purified typhoid toxin to mice is associated with lethargy, weight loss, neutropenia, and some of the neurologic symptoms associated with typhoid fever, this activity is dependent on CdtB, and not on PltA [20]. One study characterizing the activity of PltA in a tissue culture cell line [30] showed that PltA and PltB are required for activation of the DNA damage response (DDR) and the observed accumulation of cells in the G_2_/M phase, but PltA need not be catalytically active. This is not surprising given that treatment of cells with purified pertussis toxin does not result in cell death but rather in disruptions in native G-protein signaling via uncoupling of the heterotrimeric G protein complexes [80]. As the pertussis toxin’s systemic effects are what contribute to pertussis disease [21,80], it could be that PltA also contributes to the systemic, or prolonged effects of typhoid fever, but not to the development of acute disease. Therefore, while these studies suggest that the role of PltA in the procurement of the cell cycle arrest phenotype is more or less related to its ability to ensure proper trafficking of CdtB, a role for PltA in pathogenesis cannot be excluded as these studies did not characterize phenotypes that are typically associated with ADP-ribosylation. As typhoid toxin cytotoxicity is dependent on PltA and PltB [30], and studies using a mouse model to assess typhoid toxin activity in vivo have primarily used catalytically-active-PltA [76,77,81,82], it is difficult to assess any potential contributions of PltA-mediated toxicity. Drawing upon what is known about the pertussis toxin’s role in pathogenesis, it would be interesting to determine if PltA is capable of inducing histamine sensitization, lymphocytosis, and insulinemia [83], all of which are established pathologies associated with the pertussis toxin in pertussis disease [21,80].

## 4. ArtAB Toxin

Saitoh et al. first described genes of a putative bARTT in *S.* Typhimurium DT104, which they named *artAB*, for ADP-ribosylating toxin [31]. Today, at least 45 different serovars of *Salmonella* have been found, using a combination of PCR-based and in silico screening methods to detect *artAB*, 39 of which (87%) are known to also encode typhoid toxin genes (Table 2). Although the genetic regulatory machinery associated with *artAB* is not well characterized, multiple studies have found that *artAB* is encoded within a prophage. When Saitoh et al. treated cultures of *artAB*-positive *S.* Typhimurium DT104 with mitomycin C, a DNA-damaging agent that was previously associated with prophage excision [84], they observed a loss of *artAB* in these isolates [31]. Indeed, *artAB* was later characterized to be encoded within prophage Gifsy-1 in *S.* Typhimurium DT-104 isolates [85]. Interestingly, *artAB* in *S.* Inverness strain FSL R8-3668 was encoded on the prophage PhInv-1b [86], which was not homologous to Gifsy-1, suggesting that transmission of *artAB* is likely mediated by multiple prophages. Depending on the serovar, *artA* and *artB* are encoded as two discrete genes (Figure 1), or in the case of *S.* Javiana, *artA* is a pseudogene that overlaps with *artB* [81].

Given its structural homology with PtxA from the pertussis toxin (Figure 3) our understanding of ArtAB and its role in virulence has been largely guided by previous work characterizing PtxA.

### 4.1. ArtAB Toxin’s ADP-Ribosyltransferase Activity and Structure

ArtAB is composed of one subunit of ArtA that uses a pentamer of ArtB subunits for the binding domain (Figure 2) [33]. Although the crystal structure of the holotoxin has not been solved, the crystal structure of individual subunits (both known and modelled) shows an overall conserved structure of ArtA with PltA and PtxA (Figure 3). Alignment of ArtA with PtxA shows the conservation of key amino acid residues that are critical for both NAD^+^ binding and the catalytic activity of PtxA. Namely, residues Arg9 and Cys41 involved in NAD^+^ binding, and catalytic residues His35 and Glu129, involved in the mature ArtA polypeptide (i.e., following hypothetical cleavage of the signal peptide [21]) are conserved in ArtA (Figure 3).

Given ArtA’s conserved catalytic and structural amino acid residues, Uchida et al. predicted that the target of ArtA’s ADP-ribosyltransferase activity, like PtxA of the pertussis toxin, would be host G-proteins. Similar to PtxA, reduction of the disulfide bonds is necessary for ArtB activity [34]. Co-incubation of both culture supernatants of *S.* Typhimurium DT104 and ArtA expressed in *E. coli*, with CHO cell post-nuclear supernatants, identified at least one 41 kDa protein that was ADP-ribosylated [34]. Using pertussis toxin as a control, which is known to ADP-ribosylate cysteine residues at the carboxy-terminus of host G_αi2_ and G_αi3_ proteins [87], ArtA-treated lysates prevented pertussis toxin-mediated ADP-ribosylation of host proteins [34], suggesting that ArtA and PtxA ADP-ribosylate the same residues in G proteins. In vitro ADP-ribosylation of purified G_αi2_, and to a lesser extent G_αi3_, proteins, confirmed that ArtA acts in a similar manner to PtxA from the pertussis toxin [88]. Taken together, the pertussis toxin’s well-established role in virulence [80,83,89] and the parallels between ArtA and PtxA’s structure and conservation of key amino acid residues, this novel bARTT may play an important role in *Salmonella* virulence.

### 4.2. Implications of ArtAB-Mediated Salmonella Virulence

Treatment of various cell lines with purified ArtAB from *S*. Typhimurium DT104 recapitulates some of the phenotypes established for pertussis toxin cytotoxicity [80,83,90], including a cell clustering phenotype in CHO-K1 cells [34], increased levels of intracellular cAMP in isoproterenol-induced RAW 264.7 macrophage-like cells [33], increased serum insulin levels (e.g., insulinemia), and intraperitoneal injection of neonatal mice (a model system commonly used for pertussis toxin), resulted in premature death [33]. Interestingly, ArtAB from *S. bongori* showed reduced cytotoxicity in both cellular and mouse models, likely due to differences in ArtB binding affinity to host cells [33]. It is important to note that ArtAB did not increase the levels of circulating white blood cells (WBCs; leukocytosis), which is a key role of the pertussis toxin in promoting pertussis disease [21]. Future studies characterizing ArtAB’s potential role in virulence, including additional analyses, such as histamine sensitization, inflammatory histopathology, cytokine expression, and differences in CD4^+^ and CD8^+^ T cell populations, all of which have been demonstrated in mouse models of pertussis [91], are needed to better understand ArtAB’s role in virulence. As NTS serovars promote an inflammatory response in the host in order to take advantage of the production of alternate terminal electron acceptors [17], it is interesting that the pertussis toxin is associated with reductions in recruited neutrophils and decreases in pro-inflammatory cytokine production at the onset of disease, which is likely reflective of the inactivation of G-protein-coupled chemokine receptors due to ADP-ribosylation [89]. Therefore, ArtAB’s role in pathogenesis is likely dependent on *Salmonella*’s ability to both balance pro-inflammatory responses and delay *Salmonella* clearance by immune cells. Studies examining ArtAB in the context of *Salmonella* infection will be important for understanding what roles this toxin might play.

## 5. SboC/SeoC Toxin

Whole genome sequence characterization of *S. bongori* identified several novel SPI-1-encoded genes not found in *S. enterica* subsp. *enterica* strains [46]. One such gene, *sboC*, shared 57% sequence homology with EspJ from enteropathogenic *E. coli* (EPEC) and the rodent pathogen *Citrobacter rodentium* [32]. EspJ is a type III secretion effector of EPEC strains and is encoded on the cryptic prophage CP-933U [92]; EspJ simultaneously ADP-ribosylates and amidates the conserved kinase-domain residue Glu310 of Src kinase [35]. While homologues of SboC have not been identified in *S. enterica* subsp. *enterica*, screening of other *S. enterica* subspecies identified an additional homolog, called SeoC, in *S. enterica* subsp. *arizonae* isolates (8/9 isolates screened) and *S. enterica* subsp. *salamae* (4/7 isolates screened) [32,46]. Alignment of the translated amino acid sequences of SeoC from both subsp. *arizonae* (83% identity) and subsp. *salamae* (77–78% identity) strains demonstrates a high level of homology with SboC [32]. Alignment with both *E. coli* and *C. rodentium* EspJ showed 56–57% amino acid identity among SeoC/SboC from *Salmonella* [32]. Although infections with *S. bongori* and *S. enterica* subsp. *arizonae* and *salamae* are relatively rare, the discovery of SboC/SeoC is important, as it demonstrates the utility of whole genome sequencing analysis in the identification of novel virulence factors in lesser studied salmonellae.

### 5.1. SboC/SeoC Toxin’s ADP-Ribosyltransferase Activity and Structure

Given the recent discovery of these toxins, formal crystallization studies have not been performed for SboC or SeoC or its homolog EspJ, although alignment of amino acid sequences shows conservation of Arg79 and Asp187 residues involved in the catalytic function [32]. Translocation of SboC and SeoC was mediated by the T3SS encoded within *Salmonella* pathogenicity island 1, as Δ*invA* mutants failed to translocate SboC and SeoC into HeLa cells [32,46]. Interestingly, inactivation of either the SPI-2 or the locus of enterocyte effacement (LEE)-encoded T3SSs also marginally decreased translocation of SeoC, suggesting that multiple T3SSs may be involved in its translocation [32]. Upon entering the cell, SboC/SeoC ADP-ribosylates residue Glu310 of Src kinase [32] and the Glu236 residue of Csk [93], and potentially other nonreceptor tyrosine kinases.

### 5.2. Implications of SboC/SeoC-Mediated Salmonella Virulence

Although infections with *S. bongori* and *S. enterica* subspecies *arizonae* and *salamae* occur in humans [94], these salmonellae are more frequently associated with the resident microbiota of reptiles [95]. Furthermore, while *S. bongori* and *S. enterica* subspecies *arizonae* and *salamae* encode SPI-1 genes, which are essential for invasion of host cells [96], *S. bongori* and subsp. *arizonae* lack SPI-2 genes that would be necessary for intracellular survival [46], therefore decreasing the likelihood of their causing salmonellosis in healthy animals/humans. Infection of J774.A1 macrophage-like cells with *sboC/seoC*-encoding *Salmonella* reduced phagocytosis of IgG opsonized beads, suggesting that similar to EspJ, ADP-ribosylation of nonreceptor tyrosine kinases is inhibitory to opsonophagocytosis [32], which could reduce the number of bacteria phagocytized by immune cells. One study found that intestinal epithelial cells collected from C57BL/6 mice infected with wild-type *C. rodentium* had an altered proteome, having significantly lower levels of proteins involved in cytokine responses, immune cell migration and adhesion, and phagocytosis, compared to the levels of these proteins among intestinal epithelial cells of mice infected with the Δ*espJ* mutant [93]. In the context of *S. bongori* and *S. enterica* subsp. *arizonae* and *salamae*, SboC/SeoC may reduce phagocytic killing, allowing the bacteria to persist in a susceptible host, although experimental confirmation will be necessary to assert a role for SboC/SeoC.

## 6. Taking What We Know About *Salmonella* bARTTs and Moving the Field Forward

Our understanding of the potential role that toxins play in salmonellosis, including the potential for host adaptation/specificity, represents an important and exciting area of research. As novel [30,32,46] and well-characterized [51] bARTTs have been identified from genomic characterizations of more diverse *Salmonella* strains, the true diversity of *Salmonella* bARTTs is likely underestimated. One key hurdle in documenting this diversity is related to the lack of publicly available whole genome sequence data for less common *Salmonella* serovars; two recent studies used whole genome sequence data to analyze 246 [97] and 266 [98] different serovars, representing just ~15–17% of *S. enterica* subsp. *enterica* serovars [2]. With the transition from traditional typing schemes (e.g., serology, MLST, PFGE, phage typing) to whole genome sequencing [99,100], bioinformatic screens of previously un-sequenced serovars will likely reveal new serovars encoding known bARTTs as well as novel bARTTs.

Although SpvB was the first *Salmonella* bARTT to be discovered, our understanding of the mechanistic role that SpvB plays during an infection is surprisingly less studied compared to the novel bARTTs. The application of novel genetic tools and cell and animal models to revisit SpvB’s role in pathogenesis may enhance our understanding of why *spvB*-encoding serovars are typically more virulent than their *spvB*-null relatives, especially considering that a number of studies compared the phenotypes of strains before and after plasmid curing, essentially testing the cumulative effects of the entire Spv operon. Given SpvB’s limited distribution among *Salmonella* serovars and its association with serovars being more likely to cause invasive disease, an investigation into the outcomes of exposure to this toxin, both at the cellular and host levels would be beneficial in furthering our understanding of the biological significance of SpvB and other actin-targeting bARTTs during an infection. Furthermore, characterizations aimed at determining within-serovar conservation of these bARTTs would be beneficial in informing our understanding of why some serovars, and even some strains within a serovar, are more likely to infect, and/or cause disease in a given host. Last, the majority of characterizations of these toxins focused on their role in acute salmonellosis. As these bARTTs may have immunomodulatory effects (e.g., neutropenia associated with the typhoid toxin), an examination of the role(s) that bARTTs play in terms of prolonging *Salmonella* clearance from the host (i.e., prolonged shedding), and their potential role in recurrent infection as a result of dampening the adaptive immune response, presents an intriguing, and presently, unanswered question.

Overall, extensive characterizations of *Salmonella* bARTTs have either not been done, or characterizations are reflective of just a handful of serovars or strains. Of particular interest is the potential role that these bARTTs play in regard to (i) enhancing virulence of different serovars, (ii) adaptation to, or restriction to a particular host(s), and (iii) how these bARTTs could be used as potential targets of novel treatment or preventive strategies, such as has been done with pertussis toxin in the dTaP vaccine [101]. Investigation of the roles that *Salmonella* bARTTs play could also serve to clarify our understanding of the potential roles of homologous bARTTs found in other species.

## 7. Conclusions

*Salmonella* remains one of the most important bacterial pathogens worldwide. The discovery of novel bARTTs encoded by a diverse number of *Salmonella* serovars represents an exciting new avenue with the potential for uncovering both unresolved questions about differences in disease severity, and alternative strategies for reducing the morbidity and mortality associated with salmonellosis. The examination of these, and homologous bARTTs found in other bacterial pathogens, demonstrate the wide array of mechanisms that bacteria have evolved to alter host responses in order to promote their survival and transmission.

## Figures and Tables

**Table 1:** Current understanding of bacterial ADP-ribosylating toxins (bARTTs) in *Salmonella* spp.

bARTT	Host Cell Targets ^1^	Known Homologues	Known Contributions to Pathogenesis	References
ArtAB	G_αi2_ and G_αi3_	PtxA (*B. pertussis*) PltA (*S*. *enterica*)	Insulinemia Mortality in neonatal mice	[33,34]
PltA (Typhoid toxin)	Unknown	PtxA (*B. pertussis*) ArtA (*Salmonella* spp.)	Unknown	[30]
SboC/SeoC	Src kinase residue Glu310, Csk kinase residue Glu236	EspJ (*E. coli* and *C. rodentium*)	Unknown	[32,35]
SpvB	Actin monomer residue Arg177	None	Colonization of intestinal lamina propia Promotes colitis	[36,37]

^1^ The putative host cell target for ArtAB is listed [33,34], although further confirmatory analyses are still needed.

**Table 2:** Distribution of ArtAB, typhoid toxin, SboC/SeoC, and SpvB toxin genes.

Species/Sub-Species	Serovar/Species	Serogroup ^1^	# of Reported Human Cases in 2006–2016 USA ^2^	*artAB* ^3^	*pltAB* (Typhoid Toxin) ^3^	*sboC/seoC* ^3^	*spvB* ^3^	References
*S. bongori*	Multiple	Multiple	17	+	+	+/-		[46]
I (*enterica*)	Abony	O:4 (B)	62	+/-	+/-			[33,47]
	Abortusequi	O:4 (B)	5	-	-		+	[33,48,49]
	Abortusovis	O:4 (B)	0				+	[50]
	Agbeni	O:13 (G)	522	-	+			[47]
	Agoueve	O:13 (G)	37	+	-			[33]
	Alachua	O:35 (O)	179	+/-	-			[33,47]
	Arechavaleta	O:4 (B)	106	+	+			[47]
	Barranquilla	O:16 (I)	89	+	+			[47]
	Bovismorbificans	O:8 (C_2_-C_3_)	817	+/-	-		+	[33,47,51]
	Brandenburg	O:4 (B)	882	+	+			[33]
	Bredeney	O:4 (B)	351	+	+			[33]
	Chester	O:4 (B)	474	+	+			[33]
	Choleraesuis	O:7 (C_1_)	181	-	-		+	[33,47,48]
	Corvallis	O:8 (C_2_-C_3_)	261	+/-	+/-			[33,47]
	Cotham	O:28 (M)	429	+	+			[47]
	Cubana	O:13 (G)	186	-	+/-			[33,47]
	Dublin	O:9 (D_1_)	1,388	-	-		+	[33,47,48]
	Durban	O:9 (D_1_)	156	+	+			[33]
	Enteritidis	O:9 (D_1_)	83,303	-	-		+	[33,47,48,52]
	Essen	O:4 (B)	16	+	+			[33]
	Freetown	O:38 (P)	8	-	+			[33]
	Gallinarum	O:9 (D_1_)	0	-	-		+	[47,48]
	Gaminara	O:16 (I)	981	+	+			[47]
	Georgia	O:7 (C_1_)	19	+	+			[47]
	Give	O:3,10 (E_1_)	1,309	+	+			[33,47]
	Glostrup	O:8 (C_2_-C_3_)	48	+	+			[47]
	Indiana	O:4 (B)	302	+	+			[47]
	Inganda	O:7 (C_1_)	8	+	+			[33]
	Inverness	O:38 (P)	620	+	-			[47]
	Javiana	O:9 (D_1_)	25,955	+	+			[33,47,52]
	Johannesburg	O:40 (R)	434	+	+			[33,47]
	Kiambu	O:4 (B)	656	-	+			[33,47]
	Kintambo	O:13 (G)	98	-	+			[47]
	Kisarawe	O:11 (F)	27	+	+			[47]
	Luciana	O:11 (F)	61	-	+			[47]
	Miami	O:9 (D_1_)	1,203	-	+			[47]
	Minnesota	O:21 (L)	301	+	+			[33,47]
	Mississippi	O:13 (G)	5,771	+	+/-			[33,47,52]
	Montevideo	O:7 (C_1_)	11,495	+	+			[33,47,52]
	Muenster	O:3,10 (E_1_)	757	-	+/-			[33,47]
	Oranienburg	O:7 (C_1_)	8,012	+	+			[33,47,52]
	Overschie	O:51	16	+	+			[47]
	Panama	O:9 (D_1_)	1,980	+	+			[33,47]
	Paratyphi A	O:2 (A)	1,716	+	+			[47]
	Paratyphi C	O:7 (C_1_)	11	-	-		+	[47,53]
	Pomona	O:28 (M)	713	+	+			[47]
	Poona	O:13 (G)	3,844	+	+			[47]
	Reading	O:4 (B)	858	+	+			[47]
	Rubislaw	O:11 (F)	1,757	+	+			[47]
	Ruiru	O:21 (L)	7	+	+			[33]
	Sandiego	O:4 (B)	1,982	+	+			[47]
	Schwarzengrund	O:4 (B)	2,934	+	+			[33,47]
	Sendai	O:9 (D_1_)	0				+	[48]
	Strasbourg	O:9,46 (D_2_)	0	-	+			[33]
	Telelkebir	O:13 (G)	339	+	+			[47]
	Typhi	O:9 (D_1_)	4,788	+	+			[47]
	Typhimurium	O:4 (B)	63,773	+/-	-		+	[33,47,48,52]
	Urbana	O:30 (N)	511	+	+			[47]
	Wandsworth	O:39 (Q)	114	+/-	+/-			[33,47]
	Welikade	O:16 (I)	5	+	+			[33]
	Worthington	O:13 (G)	363	+/-	-			[33,47]
II (*salamae*)	Multiple	Multiple	271			+/-		[32]
IIIa (*arizonae*)	Multiple	Multiple	959			+/-	+/-	[32]

^1^ serogroup as defined by [54], including both the new (e.g., O:13) and former (e.g., “G”) naming conventions. ^2^ Number of human clinical cases reported to the CDC between 2006–2016 [55]. ^3^ Gene presence/absence is listed as “+” for present, “-” absent, or “+/-” for variably detected among strains. Blanks signify that isolates were not screened for given gene(s).
